# Postmortem transcriptional profiling reveals widespread increase in inflammation in schizophrenia: a comparison of prefrontal cortex, striatum, and hippocampus among matched tetrads of controls with subjects diagnosed with schizophrenia, bipolar or major depressive disorder

**DOI:** 10.1038/s41398-019-0492-8

**Published:** 2019-05-23

**Authors:** Thomas A. Lanz, Veronica Reinhart, Mark J. Sheehan, Stacey J. Sukoff Rizzo, Susan E. Bove, Larry C. James, Dmitri Volfson, David A. Lewis, Robin J. Kleiman

**Affiliations:** 1 Translational Biology, Biogen, Cambridge, MA UK; 2 Alzheimer’s Disease Research Unit, Biogen, Cambridge, MA UK; 3 Department of Neurobiology, University of Pittsburgh School of Medicine, Pittsburgh, PA USA; 4 Aging Institute, Department of Medicine, University of Pittsburgh School of Medicine, Pittsburgh, PA USA; 5 Life Technologies, New London, CT UK; 6 Internal Medicine, Pfizer, Cambridge, MA USA; 7 Neuroscience Analytics, Takeda Pharmaceuticals, Cambridge, MA UK; 8 Department of Psychiatry, University of Pittsburgh, Pittsburgh, PA USA

**Keywords:** Schizophrenia, Molecular neuroscience

## Abstract

Psychiatric disorders such as schizophrenia (SCZ), bipolar disorder (BD), and major depressive disorder (MDD) arise from complex interactions between genetic and environmental factors. Common genetic variants associated with multiple psychiatric disorders suggest that shared genetic architecture could contribute to divergent clinical syndromes. To evaluate shared transcriptional alterations across connected brain regions, Affymetrix microarrays were used to profile postmortem dorsolateral prefrontal cortex (DLPFC), hippocampus, and associative striatum from 19 well-matched tetrads of subjects with SCZ, BD, MDD, or unaffected controls. SCZ subjects showed a substantial burden of differentially expressed genes across all examined brain regions with the greatest effects in hippocampus, whereas BD and MDD showed less robust alterations. Pathway analysis of transcriptional profiles compared across diagnoses demonstrated commonly enriched pathways between all three disorders in hippocampus, significant overlap between SCZ and BD in DLPFC, but no significant overlap of enriched pathways between disorders in striatum. SCZ samples showed increased expression of transcripts associated with inflammation across all brain regions examined, which was not evident in BD or MDD, or in rat brain following chronic dosing with antipsychotic drugs. Several markers of inflammation were confirmed by RT-PCR in hippocampus, including S100A8/9, IL-6, MAFF, APOLD1, IFITM3, and BAG3. A cytokine ELISA panel showed significant increases in IL-2 and IL-12p70 protein content in hippocampal tissue collected from same SCZ subjects when compared to matched control subjects. These data suggest an overlapping subset of dysregulated pathways across psychiatric disorders; however, a widespread increase in inflammation appears to be a specific feature of the SCZ brain and is not likely to be attributable to chronic antipsychotic drug treatment.

## Introduction

Psychiatric disorders arise from complex interactions between genetic and environmental factors, which change developmental trajectories of circuits underpinning a continuum of neuro-functional domains^1^. Common genetic associations across diagnostic boundaries of neuropsychiatric disorders^2,3^ suggest the potential for shared elements of disease etiology. Linking genetic risk factors to cellular pathways implicated in psychiatric disease requires a better understanding of the pathways impacted in disease. One approach to identifying these molecular and biochemical differences is to examine molecular profiles of postmortem brains from subjects with clinically defined psychiatric disorders and unaffected comparison subjects. Transcriptional profiling has been used extensively to characterize postmortem brain samples from subjects with schizophrenia over the past 15 years with the overarching goal of developing molecular insight into pathophysiology and candidate pathways for therapeutic intervention. Lessons learned from more than a decade of RNA profiling have highlighted many difficulties with cross-study replication of results for individual genes stemming from a variety of technical and biological factors (reviewed in ref. ^4^). Methodological differences among studies have been driven by the evolution of transcriptional profiling platforms and the accumulated scholarship regarding the impact of biological variability stemming from differences in age, gender, pH, postmortem interval (PMI), agonal state, dissection techniques, and RNA extraction. Improvements over time have meant that most published microarray studies are difficult to combine or compare because of differences in collection and/or processing. Despite limitations of study-specific findings related to individual genes, multiple cellular pathways have been consistently represented by dysregulated genes identified across studies of schizophrenia, which fall into several broad categories including immune function, oxidative phosphorylation and mitochondrial function, synaptic transmission, and myelination/glial function. These trends have been further borne out by a recent ‘mega-analysis’ to reanalyze 19 brain-based gene expression studies^5^.

We have profiled a curated collection of brain samples from the University of Pittsburgh, designed to circumvent many of the previous confounds and to enable direct comparisons across brain regions and diagnoses. The current collection is comprised of tetrads of disease and control individuals; within each tetrad, subjects were matched for sex and as closely as possible for age. All samples have been collected from the same hemisphere using the same anatomical landmarks at the coroner’s office, with low PMI, and high pH (above 6.5) and high RNA integrity to minimize technical variability among samples. Multiple brain regions have been dissected from each individually donated brain to allow profiling across brain regions from this matched set of samples.

The present study focused on three major psychiatric disorders with overlapping symptom domains: schizophrenia (SCZ), bipolar disorder (BD) and major depressive disorder (MDD). The prefrontal cortex (PFC) has been a focus for much investigation across these disorders, given its prominent role in working memory and the integration and ordering of environmental stimuli^6–8^. A key feature of the current analysis is the profiling of PFC, hippocampus, and striatum from the same individuals. Functional connectivity between these regions is important to a variety of cognitive functions, and disruptions to this network have been implicated in psychiatric disorders^9–12^. Prior studies investigated expression of BDNF pathway transcripts using RT-PCR on this sample set revealed a striatal TrkB mRNA reduction in both BD and MDD subjects, while reductions in specific BDNF transcripts in hippocampus were observed in both SCZ and BD subjects^13^. The present further leveraged these well-matched tetrads to identify other potential common pathways among disease states or functionally connected anatomical regions. To additionally evaluate the potential contributions of antipsychotic medication to gene expression differences, we generated rat pharmacology gene expression datasets from three different brain regions following chronic treatment with haloperidol or risperidone at doses selected to produce clinically meaningful exposures. Our results highlight differences in gene expression between SCZ, BD, and MDD subjects relative to unaffected controls, with some common pathways enriched across different pairs of disorders in select brain regions. We highlight the severity of gene expression dysregulation in SCZ as compared to BD and MDD (SCZ≫BP > MDD) and the magnitude of gene expression dysregulation in the hippocampus as compared to the connected dorsolateral prefrontal cortex and striatum (hippocampus > striatum > DLPFC) from SCZ. We highlight the robust and disease-specific upregulation of cellular pathways related to inflammation across brain circuitry in SCZ subjects.

## Methods

### Postmortem human samples

Brains from tetrads of subjects with SCZ, MDD, or BD and unaffected comparison subjects were obtained at the University of Pittsburgh as described previously^14^. Subject groups did not differ in mean age, postmortem interval, brain pH, RNA integrity number (RIN) or tissue storage time (Supplementary Table 1). Cryostat sections cut from Brodmann Area 46 (dorsolateral prefrontal cortex, DLPFC), associative subregion of dorsal striatum (as described by Suzanne Haber^15^), and hippocampus were collected into empty tubes (for protein analysis) or tubes containing TRIzol (Life Technologies, Carlsbad, CA), from which RNA was extracted using a phenol-chloroform extraction method. All samples were analyzed using an Agilent Bioanalyzer (Agilent Technologies, Santa Clara, CA) to evaluate total RNA quality. RNA was converted to cDNA using Applied Biosystems High Capacity RNA-to-cDNA Master Mix (Life Technologies). RNA was hybridized to U133_Plus2 Affymetrix whole genome microarray chips.

### qRT-PCR

TaqMan assays were ordered for each gene of interest (Supplementary Table 2; Life Technologies). Additionally, 16 endogenous control genes were selected for each brain region and assessed across eight subjects per group per region. Those six most stable endogenous control genes were analyzed across all samples. The three most stable genes across all groups and regions, identified using Normfinder^16^, were selected as reference genes for normalization (RPN1, PSMB2, and RPL13A). Quantitative RT-PCR was performed on an Applied Biosystems ViiA 7 thermocycler (Life Technologies, Carlsbad, CA) in 384-well plates. Reactions were conducted in quadruplicate containing 15 ng RNA per well. Relative standard curve qPCR was used to derive quantities for the GOIs and reference genes using the ViiA 7 software. Normalized quantities (NQ) were calculated by dividing the GOI quantity by the geometric mean of the quantity of the three reference genes for each sample.

### Cytokine measurement

Brain samples were measured for cytokine levels as described previously^17^. Sections were homogenized in 150 mM NaCl, 20 mM TrisHCl pH 7.5, 1 mM EDTA, 1 mM EGTA, 1% Triton-X 100, 1X HALT protease and phosphatase inhibitor cocktail. Samples were homogenized briefly on ice, then centrifuged at 15,000 × *g* for 20 min at 4 °C. Lysates were run in triplicate in a multiplex ELISA for a panel of cytokines (Meso Scale Discovery, Rockville, MD) and values were normalized to total protein.

### Statistical analysis

Microarray data were normalized by Robust Multi-array Average (RMA), and data were deposited in GEO (GSE53987). Only genes expressed in all three regions were included in the statistical analysis (*N* = 22,461). All data (microarray, RT-PCR and cytokine) were analyzed for differential expression using a mixed model ANCOVA using log10 transformed RMA, RQ, or interpolated cytokine protein values as response variable, brain region, and disease group as fixed factors, tetrad as a random factor, and age, sex, tobacco use at time of death, manner of death (accidental, natural, or suicide), PMI, and pH as covariates. Significant ANCOVA results were followed by post-hoc comparisons between disease and control least-squares means, and Tukey–Kramer multiple comparison test. These statistical tests were performed two-tailed at 5% level of significance in R statistical software version 3.1.0 (Supplementary Table 3).

For conventional pathway analysis of microarray data, genes with >1.2-fold change and FDR-adjusted *p* < 0.05 were uploaded to Ingenuity Pathway Analysis (IPA). Significantly enriched pathways identified in IPA (*p* < 0.05) were compared between groups using Fisher’s test using *p* < 0.05 as a cutoff for enrichment of overlap (GraphPad Prism 7). Pathways categorized in IPA as “disease-associated” were excluded from analysis, as these are redundant with core signaling pathways. For separate analysis of up- and downregulated transcripts, statistically significant enrichment scores (−log(*p*-value)) for IPA pathways were compared across all conditions. Pathways whose enrichment scores corresponded to *p* < 0.05 were re-analyzed after separation into up- and downregulated genes. Enrichment scores for downregulated genes were subtracted from the enrichment scores for upregulated genes to result in net positive enrichment if upregulated genes drove enrichment, net negative genes if downregulated genes drove enrichment, and scores centered around zero had a balance of up and downregulated genes. For the top 50 pathways enriched in SCZ, heat maps were generated from the net enrichment scores in Spotfire (TIBCO, Boston, MA).

Weighted gene co-expression network analysis (WGCNA^18^) was also performed on the normalized microarray data. Signed consensus networks were constructed using the blockwise Consensus Modules function. Soft-thresholding power was set to 12, to achieve approximate scale-free topology (model fit R^2^ > 0.8) in all brain regions individually. Genes were pre-clustered by consensus Projective K Means and assigned to four blocks. Networks were constructed for each block using Pearson correlation, and topological overlap was calculated. Individual brain region topological overlap matrices (TOMs) were single quantile scaled, and for each pair of genes, the minimum topological overlap across the brain regions was retained to generate the consensus TOM. Genes were subsequently clustered using average linkage hierarchical clustering of the dissimilarity matrix (1-TOM). Modules were identified by dynamic hybrid tree cut of the dendrogram with a minimum module size of 50 and a deepsplit of 4. The first principal component of the identified modules defined the module eigengene (ME), and genes with correlation with ME < 0.3 were removed from the module. Modules with fewer than 17 genes with correlation to ME > 0.5 were disbanded. Following module processing across all blocks, genes showing correlation *p*-values to another ME lower (i.e., more significant) than that of the module to which they were assigned by a factor of 1E-4 were re-assigned to the closer module. Finally, MEs were clustered by one minus their correlation, the resulting dendrogram was cut at 0.15. All modules on resulting branches were merged, and the process was repeated until no modules merged after dendrogram cutting. Final ME-trait associations were determined by Pearson correlation. Fisher’s asymptotic *p*-value was calculated for all correlations and corrected for multiple corrections by FDR. Enrichment of differentially expressed genes within modules was assessed by Fisher’s exact test in GraphPad Prism 7.

### Rodent pharmacology

2-month-old male Sprague-Dawley rats (Charles River) were treated with 0.25 mg/kg/d haloperidol, 5 mg/kg/d risperidone or vehicle (1% acetic acid in water, pH 5) for 21 days via subcutaneous Alzet minipumps (*n* = 5 per group). Striatum, hippocampus, and frontal cortex were immediately frozen on dry ice. Plasma was processed for analysis of drug levels by LC/MS. Total RNA was extracted as described above. RNA was hybridized to rat genome 230_2 Affymetrix microarray chips (GEO GSE66277). Data were processed as described above, and comparison pathway analysis was performed in IPA alongside the human SCZ versus control contrasts.

## Results

### Cross-disorder pathway comparisons

Microarray analyses of DLPFC, hippocampus, and associative striatum from 19 matched tetrads of subjects with SCZ, BD, MDD, and unaffected comparison controls showed the greatest number of differentially expressed genes in control comparisons to SCZ (3158 total genes across regions), followed by BD (244), and then MDD (90). Within SCZ, the greatest number and most robust differentially expressed genes were observed in hippocampus (2001; ~9% of detected transcripts), followed by striatum (673; 3% of detected transcripts), with the fewest changes in DLPFC (484; 2% of detected transcripts). Numbers of up- and downregulated genes across region and subject group are shown in Supplementary Table 4; the full gene list is presented in Supplementary Table 5. Dysregulated canonical pathways were compared among SCZ, BD, and MDD for each region. Multiple pathways overlapped between pairs of disease states, but only in DLPFC and hippocampus was this overlap statistically significant. The full list of enriched pathways for each region can be found in Supplementary Table 6.

**Fig. 1:**
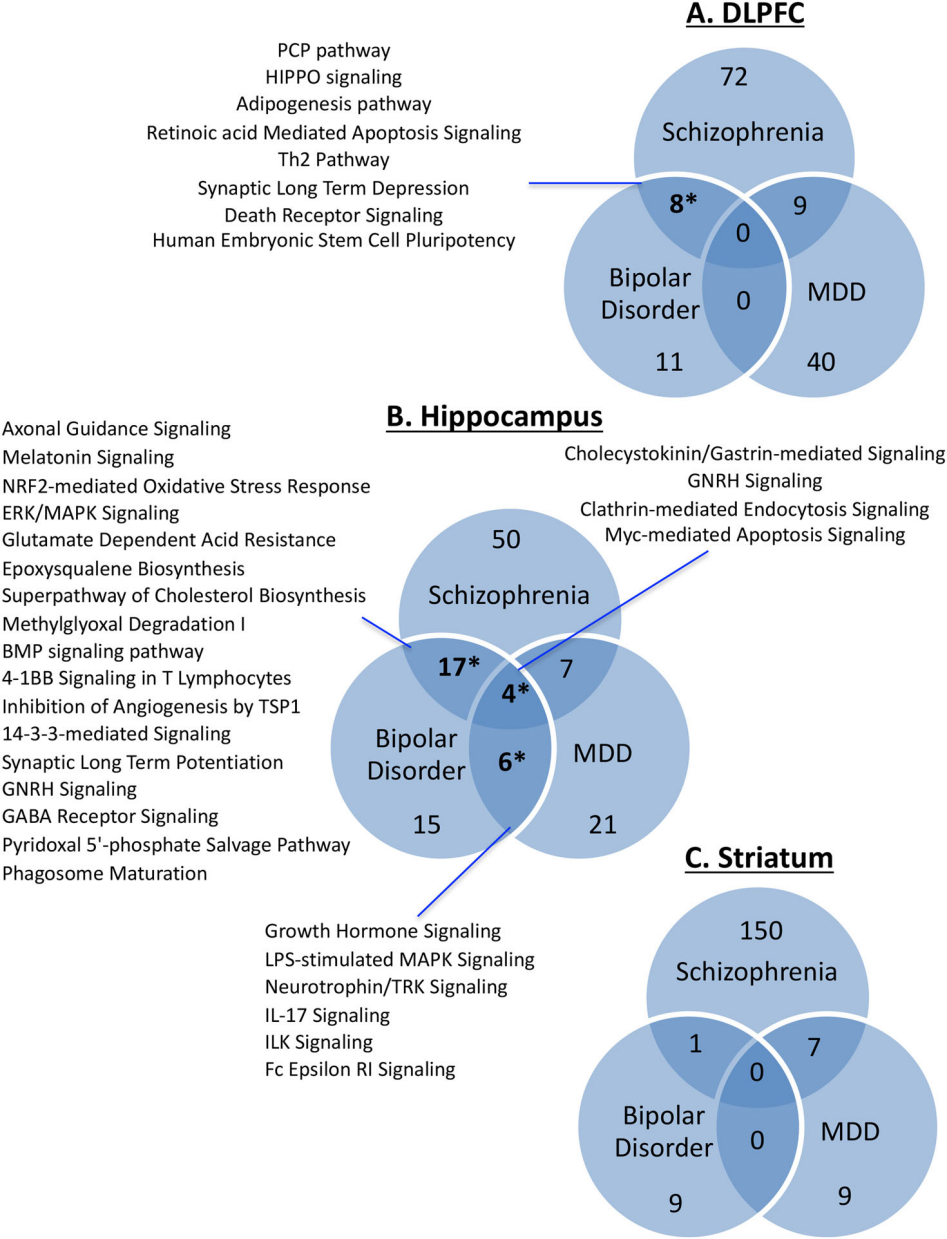
Venn diagrams for DLPFC (a), hippocampus (b) and striatum (c) representing the number of overlapping enriched pathways within each disorder. When the overlap between two disorders was statistically significant (*p* < 0.05 by Fisher’s test), the top pathways (ranked by net enrichment score) are listed alongside the diagram

There were eight pathways that showed overlap in the DLPFC between SCZ and BD (Fig. 1a), which represented a significant enrichment. These pathways included: planar cell polarity (PCP) pathway, HIPPO signaling, adipogenesis pathway, retinoic acid mediated apoptosis signaling, Th2 pathway, synaptic long-term depression, death receptor signaling, and human embryonic stem cell pluripotency. There were four pathways that showed overlap in the hippocampus among all three disease states (SCZ, BD, and MDD) (Fig. 1b). These included: cholecystokinin/gastrin-mediated signaling, gonadotropin-releasing hormone (GNRH) signaling, clathrin-mediated endocytosis signaling, and myc-mediated apoptosis signaling. Furthermore, 17 additional pathways overlapped between SCZ and BD in hippocampus, including NRF2-mediated oxidative stress response, 14-3-3 signaling, ERK/MAPK signaling, synaptic long-term potentiation, and GABA receptor signaling. Also overlapping in hippocampus between BD and MDD were six pathways: growth hormone signaling, lipopolysaccharide (LPS)-stimulated MAPK signaling, neurotrophin/tyrosine receptor kinase (TRK) signaling, IL-17 signaling, integrin-linked kinase (ILK) signaling, and Fc epsilon receptor signaling. No significant overlap was seen in any pathways across the three disorders in striatum (Fig. 1c).

All the SCZ subjects were exposed to chronic treatment with antipsychotic drugs. To evaluate the possibility that some of the pathways showing enrichment in postmortem transcriptional data reflect effects of chronic exposure to medication rather than disease biology, we conducted preclinical profiling studies. To identify potential pathways associated with exposure to common antipsychotic drugs in the brain, adult rats were dosed for 21 days with minipumps containing haloperidol or risperidone. Plasma levels of drug were measured at the end of the treatment and resulted in free plasma drug levels of 4.68 ± 0.16 nM for haloperidol and 137 ± 10.9 nM for risperidone. These concentrations represent exposures that would be expected to produce therapeutically relevant target occupancy of the D2 receptor^19^. While some pathways overlapped between the rodent antipsychotic drug treatment and SCZ postmortem brain, this overlap was not statistically significant (Supplementary Fig. 1). Many of the overlapping pathways were related to signal transduction relevant to the dopaminergic receptor, including protein kinase A signaling and CREB signaling, consistent with known mechanisms of action for these treatments. It is difficult to know whether these pathways, which were identified in both psychiatric samples and rodents subjected to chronic drug treatment, are associated with drug exposure alone or represent the profile of a therapeutic response to drug since we cannot evaluate profiles from matched unmedicated patients. Most pathways altered in patients did not show modulation by medication in rodents. Gene-level results for haloperidol and risperidone treatment are shown in Supplementary Table 7.

### Directionality of gene expression changes across regions and disorders

**Fig. 2:**
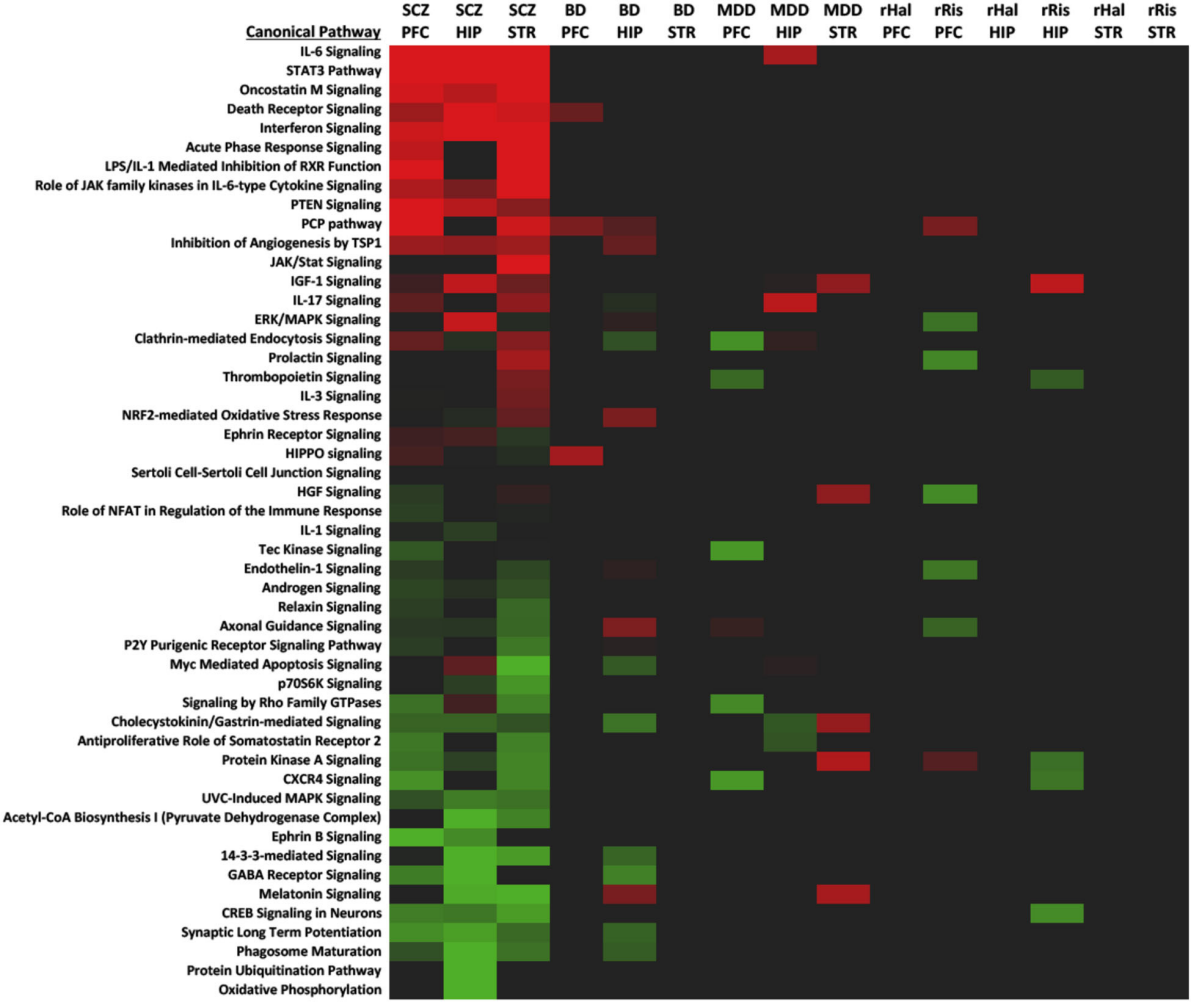
Top 50 pathways enriched in schizophrenia based on differentially expressed genes in DLPFC, hippocampus and striatum. Red = net positive score (enrichment by upregulated genes), green = net negative enrichment score (enrichment by downregulated genes), and black = no net change in expression. Schizophrenia (SCZ) scores are shown alongside scores for bipolar disorder (BD) and major depressive disorder (MDD) for DLPFC, hippocampus (HIP) and striatum (STR). The last six columns represent enrichment scores for frontal cortex (PFC), hippocampus (HIP), and striatum (STR) from rats treated for 21 days with haloperidol (rHal) or risperidone (rRis)

Both the number of gene pathways and the magnitude of gene dysregulation were enriched in SCZ compared to BD or MDD. To better understand the impact of regional gene dysregulation in SCZ, additional analysis was performed to look at the direction of these effects. The IPA analysis was run separately for up- vs downregulated transcripts in each group. The top 50 enriched pathways in SCZ are shown as a heat map in Fig. 2. This analysis demonstrates that the majority of the signaling pathways enriched in SCZ were downregulated, with oxidative phosphorylation being the most dominant, particularly in hippocampus. A variety of signaling pathways showed downregulation in multiple regions in SCZ, such as synaptic long-term potentiation, 14-3-3 signaling, CXCR4 signaling, and many others. Select pathways such as GABA receptor signaling showed similar enrichment in hippocampus for both SCZ and BD, while many other downregulated pathways appeared to be specific to SCZ. Downregulation of several GABAergic transcripts was confirmed by qRT-PCR, including SST in both BD and SCZ, and GAD1, GAD2, PVALB reductions specific to SCZ (Supplementary Fig. 2). Of note, the reductions in GABAergic transcripts were restricted to hippocampus and DLPFC; no GABAergic transcripts examined were altered in striatum, which is of interest because it is comprised largely of GABAergic medium spiny neurons.

SCZ samples showed a robust enrichment in upregulated inflammatory pathways across all brain regions examined. While some of the inflammatory pathways share common genes, such as SHC1, STAT3, and AKT2, most of the pathway enrichment was driven by distinct sets of genes in different brain regions, rather than multiple pathways implicated by a set of common core genes. Interestingly, only a few pathways, such as IL-6 signaling and STAT3 pathways, showed any enrichment in the PFC and STR of BD samples, although it was not as robust as the enrichment in SCZ. There was no evidence of enrichment of inflammatory pathways in tissue from subjects with MDD. These inflammatory pathways were also not enriched by the rodent antipsychotic treatments, although selected signaling pathways, such as long-term potentiation, PKA, and cAMP, were downregulated in both datasets. This suggests that the increase in inflammatory gene expression is more likely the result of region-specific disease processes than of drug treatment.

### WGCNA analysis

Weighted gene co-expression network analysis (WGCNA^18^) was used to further analyze the data from all human samples. This analysis defined 39 consensus modules present in all brain regions ranging in size from 60 to 1693 genes (Supplementary Table 8). Seventeen of these modules showed a significant eigenvector correlation with SCZ (Supplementary Table 9). Five of these SCZ modules were also significantly enriched for genes that were differentially expressed in SCZ relative to unaffected comparison subjects (Table 1). The largest of these networks (turquoise) showed significant negative correlation with SCZ in all three brain regions and was dominated by markers of GABAergic interneurons (e.g., NPY) and genes involved in mitochondrial function (e.g., ATP5O and ATP6V1A). A second module, yellow, was also negatively correlated with SCZ, and included additional GABAergic transcripts such as SST, as well as several genes involved in vesicle trafficking and GPCR signaling. The third module negatively correlated with SCZ, lightcyan, was enriched for DNA or RNA binding genes. Among the modules positively correlated with SCZ, the darkred module was enriched for signaling pathways that included actin cytoskeleton signaling and processes related to cell cycle, while the paleturquoise module was heavily enriched with genes involved in inflammation. We sought to corroborate these findings with direct measures of mRNA and protein in replicate hippocampal samples.

**Table 1:** WGCNA modules enriched in schizophrenia

Module	Genes in module	Differentially expressed genes			SCZ	Themes	Representative genes
		SCZ	BD	MDD	Correlation		
Turquoise	1693	726***	47**	4	−	Mitochondrial function, GABA	ATP5O, ATP6V1A, NPY
Paleturquoise	122	50***	5	1	**+**	Inflammation	HILPDA, MAFF, SOCS3
Yellow	968	268***	22	1	−	Vesicle trafficking, GPCR signaling, GABA	RAB3C, SNAP25, SST
Darkred	175	43*	0	1	**+**	Actin cytoskeleton signaling, cell cycle	ARHGEF1, CDK13, FYN
Lightcyan	231	54*	0	0	−	DNA/RNA binding	CAND1, CNOT7, TMX4

For each module, total number of genes in the module is given, followed by the number of differentially expressed genes in schizophrenia within that module. All five modules showed significant Pearson correlation with schizophrenia; positive versus negative correlations are noted with + or – symbols, respectively. Top pathways or GO terms enriched by the genes in each module are summarized in “Themes.” Representative genes (significant differential expression in schizophrenia and high connectivity scores) are listed in the last column

Asterisks denote statistically significant enrichment of differentially expressed genes, *p < 0.05, **p < 0.01, ***p < 0.001

### qRT-PCR and protein follow-up of inflammatory targets in the hippocampus

**Fig. 3:**
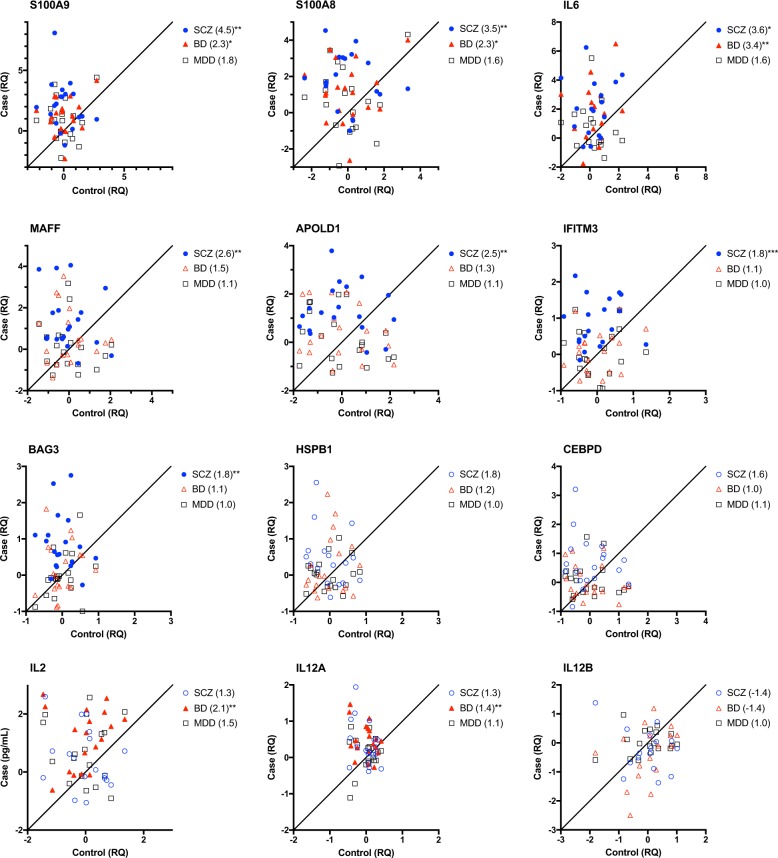
Inflammatory transcripts in hippocampus from subjects with SCZ (blue circles) BD (red triangles), or MDD (black squares) are plotted in a case-control fashion for matched pairs. Values are normalized to the geometric mean of each control group and plotted in log2 scale. Filled symbols indicated FDR-adjusted *p* < 0.05. Fold change relative to control is shown in parentheses in the legend. *p < 0.05, **p < 0.01, ***p < 0.001

Upregulation of genes in SCZ related to inflammation was a major feature using both WGCNA and Ingenuity pathway analysis. Transcripts representing 12 cytokines and effector molecules involved in inflammatory responses were selected for qRT-PCR validation in hippocampus (Fig. 3). In SCZ versus control subjects, S100A9 (14.2-fold, *p* = 0.001), S100A8 (3.6-fold, *p* = 0.003), IL6 (6.9-fold, *p* = 0.042), APOLD1 (2.3-fold, *p* = 0.004), IFITM3 (1.9-fold, *p* < 0.001), and BAG3 (2.1-fold, *p* = 0.002) transcripts were significantly higher in hippocampus following multiple testing adjustment. Expression levels of S100A9 (2.7-fold, *p* = 0.037), S100A8 (3.6-fold, *p* = 0.002), IL6 (6.9-fold, *p* = 0.009), IL2 (2.4-fold, *p* = 0.002), and IL12A (1.4-fold, *p* = 0.003) were significantly higher in BD subjects compared to control subjects. By contrast, there were no significant differences in any of the inflammatory genes tested in MDD subjects.

**Fig. 4:**
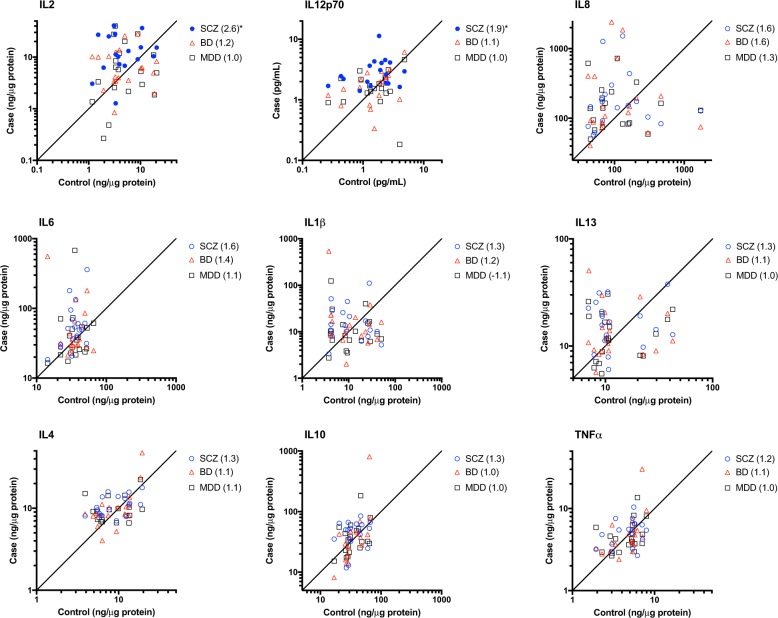
Measurement of cytokine protein levels in hippocampus from subjects with bipolar disorder (red), MDD (green), schizophrenia (blue), or unaffected comparison subjects (gray). SCZ (blue) BD (black), or MDD (grey) are plotted in a case-control fashion for matched pairs. Values are based on standard curves and given in log 2 scale (ng cytokine per μg total protein). Filled symbols indicated FDR-adjusted *p* < 0.05. Fold change relative to control is shown in parentheses in the legend

To confirm inflammatory changes at the protein level, a panel of cytokines was measured in hippocampus from all subjects by multiplex ELISA (Fig. 4). As with mRNA, brain pH was a significant covariate for IL10 and IL1B (Supplementary Table 3). PMI was a significant covariate for IL1B. Gender was determined to be a significant covariate for IL8, TNF, and IL12p70. Protein levels of IL-2 (2.52 fold, *p* = 0.021) and IL-12p70 (1.71-fold, *p* = 0.016) were significantly higher in subjects with SCZ relative to control subjects (Fig. 4). There were no significant differences in protein level for any of the cytokines tested in the hippocampus of BD or MDD patients when compared to control.

## Discussion

Dysfunction within and among the circuitry containing DLPFC, hippocampus, and associative striatum has been described for multiple psychiatric disorders^10–12^. The present study demonstrates profound dysregulation of gene expression across all three regions in subjects diagnosed with SCZ compared with control subjects, and is distinguished from BD and MDD by the magnitude of differential expression and the affected gene pathways. The greatest regional burden of transcriptional dysregulation was different for each disorder. MDD samples exhibited more differentially expressed transcripts in striatum and hippocampus than in DLPFC, whereas very few genes were altered in BD striatum. The most profound changes were detected in SCZ across all regions studied as compared with BD and MDD. Between regions, hippocampus showed the greatest number of differentially expressed genes in SCZ, while the DLPFC, which has been the focus of most previous transcriptional profiling studies, showed the fewest changes. A milder transcriptional profile phenotype in the DLPFC of subjects with SCZ is consistent with two other studies of independent patient cohorts that examined DLPFC alongside either hippocampus or temporal cortex and found evidence of greater transcriptional dysregulation in each of these areas compared with DLPFC from the same subjects^20,21^.

Previous microarray-based interrogations of prefrontal cortex in SCZ subjects have highlighted altered expression of a number of pathways relevant to synaptic function^22–24^, as well as some ubiquitous signaling pathways such as 14-3-3 signaling^25^. The present study detected significant enrichment in 14-3-3 signaling and several synaptic pathways such as LTP and GABA receptor signaling, as well as downstream effectors of synaptic function such as PKA, CREB, and MAPK signaling. Many of these pathways were also observed in BD, and in general, transcripts significantly altered in these pathways were downregulated in SCZ or BD relative to control subjects. While some of these signaling pathways may be the result of ongoing disease or pharmacologic processes, it is possible that persistent changes in certain pathways were always present and could be related to disease etiology. For example, CXCR4 signaling, which was one of the downregulated pathways in SCZ (Fig. 2), plays a critical role in interneuron migration during development^26^. As a downstream target of TBX1^27^, reduced CXCR4 has been implicated in disrupted interneuron migration in a mouse model of 22q11 syndrome^28^, which has a high burden of SCZ and other psychiatric disorders^29^. Knocking out CXCR4 specifically in parvalbumin neurons was shown to produce a stereotypy phenotype in mice^30^. While CXCR4 itself was unchanged in the present dataset, the pathway was implicated by reduced expression downstream signaling genes and its ligand CXCL12, which has also been shown to exhibit reduced expression in olfactory neurons from SCZ relative to control subjects^31^. A large qPCR analysis of postmortem prefrontal cortex measured a small elevation in CXCR4 mRNA in SCZ relative to control subjects, which was hypothesized to represent a compensatory mechanism intended to correct developmental deficits in interneuron migration^32^.

The current data are consistent with previous literature describing changes in transcriptional profiles of GABA interneurons in SCZ. GAD67 mRNA and protein reductions in DLPFC have been reproducibly reported across studies comparing SCZ subjects to unaffected controls^33–37^. Lower abundance of transcripts specific to different GABAergic interneuron populations, namely parvalbumin (PVALB), somatostatin (SST), and neuropeptide Y (NPY), have also been observed in DLPFC^35,38–41^. The present study also showed trends toward lower GABAergic transcripts in SCZ DLPFC; however, larger and more statistically significant differences in GABAergic transcripts were observed in the hippocampus from the same subjects diagnosed with SCZ, with smaller trends observed for BD. GABAergic markers were represented in two of the five WGCNA modules that were both correlated with SCZ and enriched for SCZ differentially expressed genes. One of these modules also included several genes involved in vesicle trafficking. A consequence of reduced GAD67 in interneurons would be reduced synthesis, and thus also release, of GABA. Genetic downregulation of GAD67 in mice was shown to reduce GABA release probability and produce a variety of behavioral alterations, including a deficit in paired pulse inhibition^42^. In striatum, however, no changes in GABAergic transcripts were identified in SCZ or BD. This regional difference suggests a functional distinction in the GABAergic deficit that is not intrinsic to GABAergic neurons, per se. Given that striatum contains largely GABAergic projection neurons in the form of medium spiny neurons, the altered GABAergic transcripts in SCZ and BD are seen only in DLPFC and hippocampus, where GABAergic neurons are interneurons.

The most striking result of pathway analyses carried out using the current dataset was a consistent upregulation of inflammatory pathways across all three brain regions examined in the SCZ subjects. This contrasts with downregulated changes in GABAergic and other signaling pathways that were preferentially observed in hippocampus. BD and MDD samples showed very little enrichment in the same inflammatory pathways. Previous microarray studies have highlighted inflammatory gene changes in independent cohorts of SCZ DLPFC and hippocampus^43–46^. Two recent RNAseq studies similarly emphasized inflammation as a major theme in the SCZ DLPFC^47,48^. We confirmed the observed elevations of transcripts encoding multiple inflammatory mediators using qRT-PCR, and performed a multiplex ELISA assay to demonstrate increases in cytokine protein levels of samples from SCZ hippocampus. Evidence of inflammatory dysregulation across psychiatric symptom domains has triggered significant interest in evaluating the immunomodulatory potential of psychotropic medications; however, meta-analysis of the literature in this field has not yielded consensus on the effects of antipsychotics, with methodological differences among studies yielding opposing effects for the same medication across studies^49^. Our microarray analyses of rodents dosed chronically with antipsychotic drugs showed very few overlapping inflammatory genes or pathways with SCZ, suggesting the observed enrichment is not linked to medication (with the caveat that the present analysis is limited to risperidone and haloperidol). A second caveat to the rodent data is that although the brain regions evaluated in rodents were roughly similar to those evaluated in human, there is not a direct correlate to DLPFC in rodents, and whole striatum was collected in rats, whereas associative striatum was isolated in humans. Finally, the compounds were administered to otherwise naïve rats without a neurodevelopmental or other such deficit. Nonetheless, previous studies of unmedicated SCZ subjects have also demonstrated increased inflammatory transcripts^46^. Abundant epidemiologic evidence links in utero infection and inflammation with increased risk of developing SCZ (reviewed in ref. ^50^), suggesting that increased inflammation may precede pathophysiology observed in adult patients. Animal models of prenatal immune activation produce a number of deficits relevant to SCZ, including parvalbumin reductions, working memory disruption, altered NMDA and dopamine signaling, and disrupted pre-pulse inhibition^51,52^. Furthermore, some studies show that animals exposed to an acute prenatal immune activation show persistent increases in cytokine expression in the frontal cortex and hippocampus as young adults^53,54^. Another study showed increased cytokine and inflammatory gene expression in PFC from adult mice treated with poly I:C, but not in offspring exposed to prenatal poly I:C^55^.

It is possible that the inflammation observed in postmortem SCZ brain is not simply a downstream result of disease processes, but could itself be a mediator of pathology. A potential causal role for altered immune function is suggested by the finding that the most significant GWAS association with SCZ lies within the major histocompatibility complex locus^56^. The role of microglia and complement in synaptic pruning have been suggested as a potential mechanism by which the immune system can alter the normal developmental trajectory of key circuits in the brain^57,58^. Persistent inflammation in the adult brain may further aggravate a downstream set of pathologies in vulnerable cell populations, such as fast-spiking parvalbumin (PVALB) interneurons. Work by Behrens et al. have shown that NMDA receptor inhibition leads to IL-6 mediated superoxide production in PVALB interneurons, resulting in reduced expression of PVALB and GAD67 in this cell population^59,60^. Previously, brain-specific IL-6 over-expression in transgenic mice has been shown to cause reduced PVALB expression in the neocortex and hippocampus, with a functional consequence of increasing seizure liability^61^. NMDA receptor hypofunction has been postulated as a contributor to SCZ etiology^62,63^. Additional data supporting interactions between NMDA hypofunction, inflammation and oxidative stress in SCZ have been described, with PVALB interneurons as a vulnerable target central to these interactions^64^. It should be noted that the present WGCNA included a co-expression module associated with SCZ that included a mix of mitochondrial transcripts and GABAergic transcripts, perhaps suggesting a correlative deficit. In the pathway analysis of SCZ, the oxidative phosphorylation pathway demonstrated the strongest enrichment score from down regulated transcripts, and IL-6 signaling was the strongest upregulated pathway. These findings were specific to SCZ, with minimal overlap in BD or MDD, and no representation in the preclinical antipsychotic drug datasets. These findings are also consistent with previous microarray analyses that found reduced expression of mitochondrial genes in SCZ^22,23,65^ and suggest the presence of oxidative stress in addition to inflammation in the SCZ brain.

An intriguing question arising from these data is whether treating neuroinflammation in SCZ would result in reduced oxidative stress and improve the health of PVALB interneurons. The present rodent antipsychotic data suggest that current pharmacology does not affect inflammatory pathways or oxidative phosphorylation; however, this may be due to a lack of inflammatory state in the normal, non-diseased rodents prior to treatment with the antipsychotics. Trials of existing non-steroidal anti-inflammatory drugs and similar classes of drugs have shown mixed benefit in subjects with SCZ^66^. Most current anti-inflammatory drugs, however, lack central exposure. Thus, a brain-penetrant drug aimed at reducing neuroinflammation or oxidative stress may be a viable therapeutic strategy for treating SCZ. Earlier intervention is likely to be more effective for any disease-modifying mechanism, though the persistence of inflammation in the adult SCZ brain leaves open a possibility that intervention at any time might produce measurable improvements.

## Supplementary information

Supplemental Figure and Table Legends

Supplemental Table 1

Supplemental Figure 1

Supplemental Figure 2

Supplemental Table 2

Supplemental Table 3

Supplemental Table 4

Supplemental Table 5

Supplemental Table 6

Supplemental Table 7

Supplemental Table 8

Supplemental Table 9
