# Supplementary material for: Postmortem transcriptional profiling reveals widespread increase in inflammation in schizophrenia: a comparison of prefrontal cortex, striatum, and hippocampus among matched tetrads of controls with subjects diagnosed with schizophrenia, bipolar or major depressive disorder
Source: Transl Psychiatry. 2019 May 23;9:151. doi: 10.1038/s41398-019-0492-8 (PMC6533277; doi:10.1038/s41398-019-0492-8)
Supplement: Supplementary file 1 — Supplemental Figure and Table Legends [file 41398_2019_492_MOESM1_ESM.docx]

**Supplementary Figures & Tables**

**Supplementary Figure 1.** Venn diagrams represent the number of enriched pathways in SCZ DLPFC and rat PFC following chronic treatment with risperidone or haloperidol. Significant overlapping pathways are listed in the accompanying box.

**Supplementary Figure 2.** GABAergic transcripts in DLPFC, hippocampus and striatum from subjects with bipolar disorder (red), MDD (green), schizophrenia (blue), or unaffected comparison subjects (grey). Values are normalized to the median of each control group and plotted in log2 scale. FDR adjusted p-values: #p<0.1, *p < 0.05.

**Supplementary Table 1.** Human subject and sample information. Values are mean ± standard deviation. PMI = post-mortem interval. RIN = RNA Integrity Number. Tobacco use at time of death (ATOD): Y = yes, N = no, U = unknown. MOD = Manner of Death (N = natural; A = accidental; S = suicide).

**Supplementary Table 2.** TaqMan assay information. HUGO gene ID is shown in the first column, followed by TaqMan assay ID, full gene name, chromosomal location, and exonic region targeted by the probe set.

**Supplementary Table 3.** ANCOVA and least-square means (LSM) tables for all RT-PCR and cytokine data. The p-values shown in the last column of the LSM tables are FDR-adjusted values.

**Supplementary Table 4.** Numbers represent genes with FDR-adjusted p-value <0.05 and ≥1.2-fold difference from control subjects. PFC = Brodmann area 46, HIP = hippocampal formation, STR = associative striatum.

**Supplementary Table 5.** Differentially expressed probesets (p<0.05, >1.2-fold change) for DLPFC, hippocampus and associative striatum from subjects with schizophrenia, bipolar disorder or MDD.

**Supplementary Table 6.** Full list of significantly enriched pathways in each region in schizophrenia, bipolar disorder and MDD.

**Supplementary Table 7.** Differentially expressed genes in rats treated with haloperidol or risperidol.

**Supplementary Table 8.** Gene membership for modules identified by WGCNA.

**Supplementary Table 9.** Correlation of different WGCNA modules with disease status.
