# Supplementary material for: Postmortem transcriptional profiling reveals widespread increase in inflammation in schizophrenia: a comparison of prefrontal cortex, striatum, and hippocampus among matched tetrads of controls with subjects diagnosed with schizophrenia, bipolar or major depressive disorder
Source: Transl Psychiatry. 2019 May 23;9:151. doi: 10.1038/s41398-019-0492-8 (PMC6533277; doi:10.1038/s41398-019-0492-8)
Supplement: Supplementary file 2 — Supplemental Table 1 [file 41398_2019_492_MOESM2_ESM.docx]

**Supplementary Table 1.**

|  | Control | Bipolar | Major Depressive Disorder | Schizophrenia |
| --- | --- | --- | --- | --- |
|  |  |  |  |  |
|  |  |  |  |  |
| Sex | 10 M, 9 F | 10 M, 9 F | 10 M, 9 F | 10 M, 9 F |
| Race | 18 W, 1 B | 19 W, 0 B | 18 W, 1 B | 13 W, 6 B |
| Age (years) | 48.1 ± 10.6 | 46.3 ± 9.5 | 45.2 ± 10.1 | 45.1 ± 8.5 |
| PMI (hours) | 19.5 ± 5.1 | 21.3 ± 6.6 | 20.1 ± 6.0 | 20.1 ± 6.9 |
| Brain pH | 6.6 ± 0.2 | 6.6 ± 0.2 | 6.6 ± 0.2 | 6.4 ± 0.4 |
| RIN | PFC 7.8 ± 0.6  HPC 6.4 ± 0.5  STR 8.3 ± 0.7 | PFC 7.6 ± 0.6  HPC 6.3 ± 0.7  STR 8.1 ± 0.9 | PFC 7.7 ± 0.5  HPC 6.3 ± 0.4  STR 8.3 ± 0.8 | PFC 7.7 ± 0.7  HPC 6.0 ± 0.6  STR 8.0 ± 0.8 |
| Tobacco ATOD | 5 Y, 14 N | 9 Y, 5 N, 5 U | 7 Y, 12 N | 12 Y, 7 N |
| MOD | 15 N, 4 A, 0 S | 6 N, 5 A, 8 S | 9 N, 3 A, 7 S | 8 N, 4 A, 7 S |
