# Supplementary figures and images for: Postmortem transcriptional profiling reveals widespread increase in inflammation in schizophrenia: a comparison of prefrontal cortex, striatum, and hippocampus among matched tetrads of controls with subjects diagnosed with schizophrenia, bipolar or major depressive disorder
Source: Transl Psychiatry. 2019 May 23;9:151. doi: 10.1038/s41398-019-0492-8 (PMC6533277; doi:10.1038/s41398-019-0492-8)

## A. DLPFC

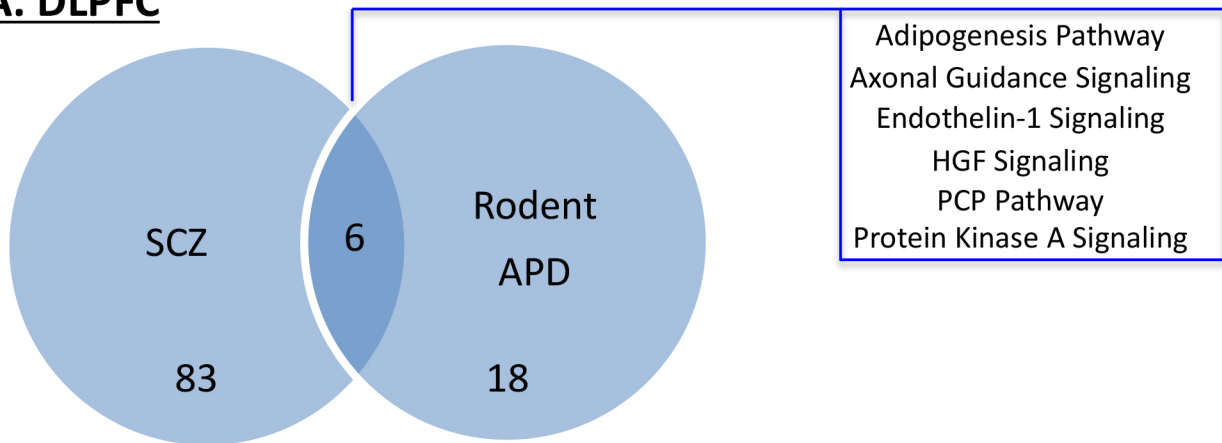

## B . Hippocampus

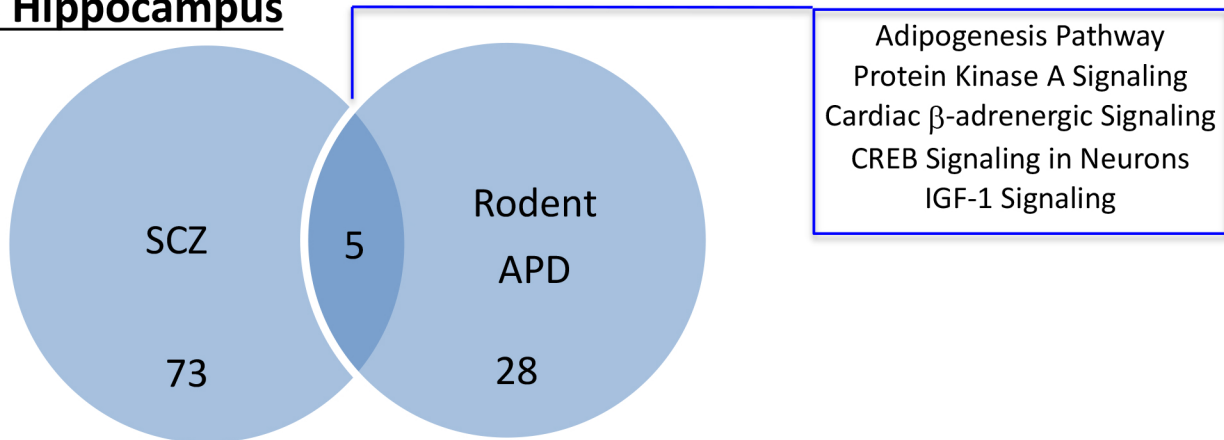

## C . Striatum

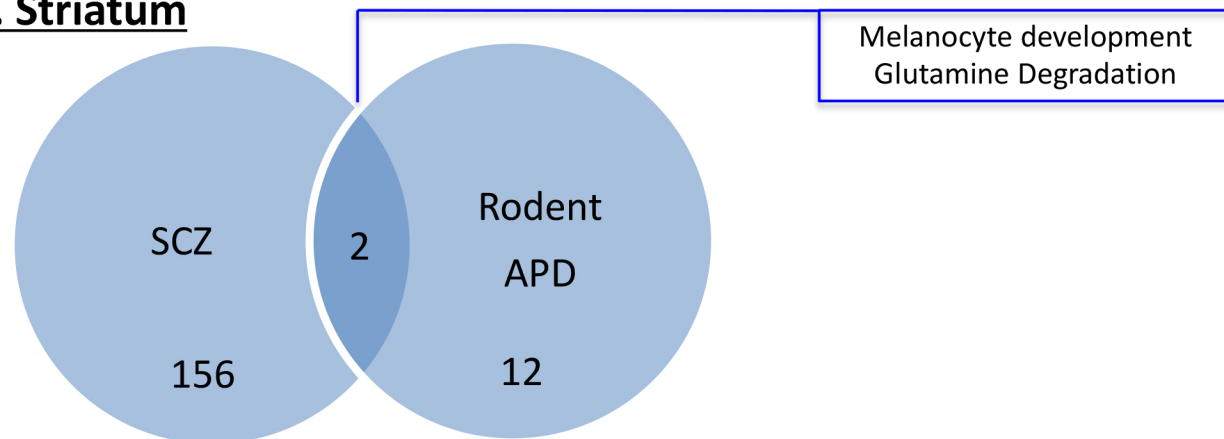

Supplement: Supplementary file 3 — Supplemental Figure 1 [file 41398_2019_492_MOESM3_ESM.pdf]
