# Supplementary material for: Postmortem transcriptional profiling reveals widespread increase in inflammation in schizophrenia: a comparison of prefrontal cortex, striatum, and hippocampus among matched tetrads of controls with subjects diagnosed with schizophrenia, bipolar or major depressive disorder
Source: Transl Psychiatry. 2019 May 23;9:151. doi: 10.1038/s41398-019-0492-8 (PMC6533277; doi:10.1038/s41398-019-0492-8)
Supplement: Supplementary file 4 — Supplemental Figure 2 [file 41398_2019_492_MOESM4_ESM.pdf]

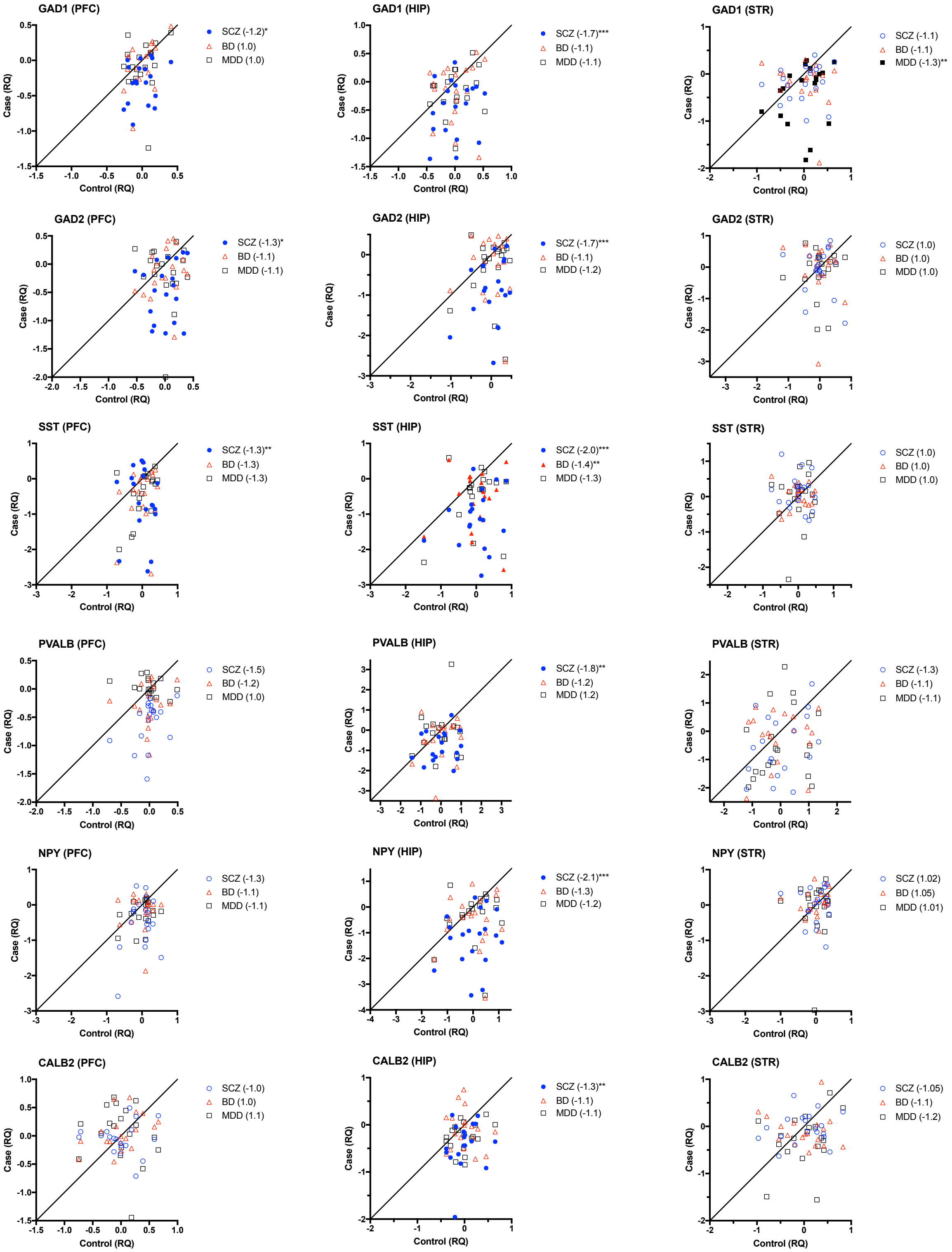
GAD1 (PFC)
SCZ (-1.2)*
BD (1.0)
MDD (1.0)
GAD1 (HIP)
SCZ (-1.7)***
BD (-1.1)
MDD (-1.1)
GAD1 (STR)
SCZ (-1.1)
BD (-1.1)
MDD (-1.3)**
GAD2 (PFC)
SCZ (-1.3)*
BD (-1.1)
MDD (-1.1)
GAD2 (HIP)
SCZ (-1.7)***
BD (-1.1)
MDD (-1.2)
GAD2 (STR)
SCZ (1.0)
BD (1.0)
MDD (1.0)
SST (PFC)
SCZ (-1.3)**
BD (-1.3)
MDD (-1.3)
SST (HIP)
SCZ (-2.0)***
BD (-1.4)**
MDD (-1.3)
SST (STR)
SCZ (1.0)
BD (1.0)
MDD (1.0)
PVALB (PFC)
SCZ (-1.5)
BD (-1.2)
MDD (1.0)
PVALB (HIP)
SCZ (-1.8)**
BD (-1.2)
MDD (1.2)
PVALB (STR)
SCZ (-1.3)
BD (-1.1)
MDD (-1.1)
NPY (PFC)
SCZ (-1.3)
BD (-1.1)
MDD (-1.1)
NPY (HIP)
SCZ (-2.1)***
BD (-1.3)
MDD (-1.2)
NPY (STR)
SCZ (1.02)
BD (1.05)
MDD (1.01)
CALB2 (PFC)
SCZ (-1.0)
BD (1.0)
MDD (1.1)
CALB2 (HIP)
SCZ (-1.3)**
BD (-1.1)
MDD (-1.1)
CALB2 (STR)
SCZ (-1.05)
BD (-1.1)
MDD (-1.2)
Case (RQ)
Control (RQ)
