# Supplementary material for: Postmortem transcriptional profiling reveals widespread increase in inflammation in schizophrenia: a comparison of prefrontal cortex, striatum, and hippocampus among matched tetrads of controls with subjects diagnosed with schizophrenia, bipolar or major depressive disorder
Source: Transl Psychiatry. 2019 May 23;9:151. doi: 10.1038/s41398-019-0492-8 (PMC6533277; doi:10.1038/s41398-019-0492-8)
Supplement: Supplementary file 7 — Supplemental Table 4 [file 41398_2019_492_MOESM7_ESM.docx]

| **Upregulated** | **Schizophrenia** | **Bipolar** | **MDD** |
| --- | --- | --- | --- |
| **PFC** | 247 | 58 | 4 |
| **HIP** | 492 | 46 | 17 |
| **STR** | 235 | 7 | 31 |
|  |  |  |  |
| **Downregulated** | **Schizophrenia** | **Bipolar** | **MDD** |
| **PFC** | 237 | 11 | 10 |
| **HIP** | 1509 | 113 | 21 |
| **STR** | 438 | 9 | 7 |
